# Unprecedented guidewire entrapment salvaged by combined rotational atherectomy and ‘Twist-wire’ technique: a case report

**DOI:** 10.1093/ehjcr/ytae258

**Published:** 2024-05-20

**Authors:** Siu-Fung Wong, Hiu-Cheong Chow, Tak-Shun Chung

**Affiliations:** Division of Cardiology, Department of Medicine & Geriatrics, United Christian Hospital, 130 Hip Wo Street, Kwun Tong, Kowloon, Hong Kong SAR, China; Division of Cardiology, Department of Medicine & Geriatrics, United Christian Hospital, 130 Hip Wo Street, Kwun Tong, Kowloon, Hong Kong SAR, China; Division of Cardiology, Department of Medicine & Geriatrics, United Christian Hospital, 130 Hip Wo Street, Kwun Tong, Kowloon, Hong Kong SAR, China

**Keywords:** Guidewire entrapment, Rotational atherectomy, Twist-wire, Case report

## Abstract

**Background:**

Coronary guidewire entrapment is not an uncommon complication of percutaneous coronary intervention, especially in the setting of complex coronary anatomy. Core wire fracture with uncoiling of spring wire represents a catastrophic complication, posing great technical difficulty in percutaneous retrieval.

**Case summary:**

The patient was a 50-year-old Asian male with ischaemic cardiomyopathy and severe left ventricular impairment. Coronary angiography showed severe left main and triple-vessel disease. Coronary artery bypass graft was declined due to high surgical risk. Percutaneous coronary intervention was performed under mechanical circulatory support. However, it was complicated with guidewire entrapment and unravelling with deformity of the newly implanted stent in the left anterior descending artery. The complication was successfully bailed out by rotational atherectomy and the novel intravascular ultrasound (IVUS) and enhanced stent visualization (ESV) system guided ‘Twist-wire’ technique. Complete wire fragments retrieval was achieved with excellent final angiographic and IVUS results immediately after procedure and at 4-month follow-up angiography.

**Discussion:**

This case represents a rare phenomenon of branch point protrusion of stent causing guidewire-stent edge entanglement. A novel ‘Twist-wire’ technique with IVUS and ESV guidance was highlighted to allow successful retrieval of fluoroscopically invisible uncoiled wire filaments.

**Learning points**

- Twist-wire technique, with the aid of IVUS and ESV system, is useful in retrieval of uncoiled fluoroscopically invisible guidewire filaments.
- Branch point protrusion is a potential pitfall in stenting of Medina 1,0,0 bifurcation lesion that is difficult to detect even with intracoronary imaging and requires a high index of suspicion for diagnosis.

## Introduction

Coronary guidewire entrapment and fracture are serious complications of percutaneous coronary intervention (PCI) that can potentially result in devastating consequences especially when the guidewire unravels.^1^ Multiple mechanisms have been proposed for the occurrence of the complication including wire jailing in bifurcation, aggressive wire manipulation including knuckling and over-rotation, severe lesion calcification, etc. The usual methods of bailout, when the wire is intact, is to advance a microcatheter or small balloon over the entrapped guidewire, with an aim to expand the ‘peri-wire’ space and allow more focused application of the withdrawal force to facilitate withdrawal.^2^ The difficulty of retrieval increases substantially in the setting of wire fracture and unravelling, which often necessitates surgical retrieval or additional stenting over the wire fragments in case of failed retrieval.^2–4^ We reported a case of unusual guidewire entrapment and unravelling that was retrieved percutaneously with a novel technique involving guidance with intracoronary imaging and stent enhancement system.

## Summary figure

**Figure:**
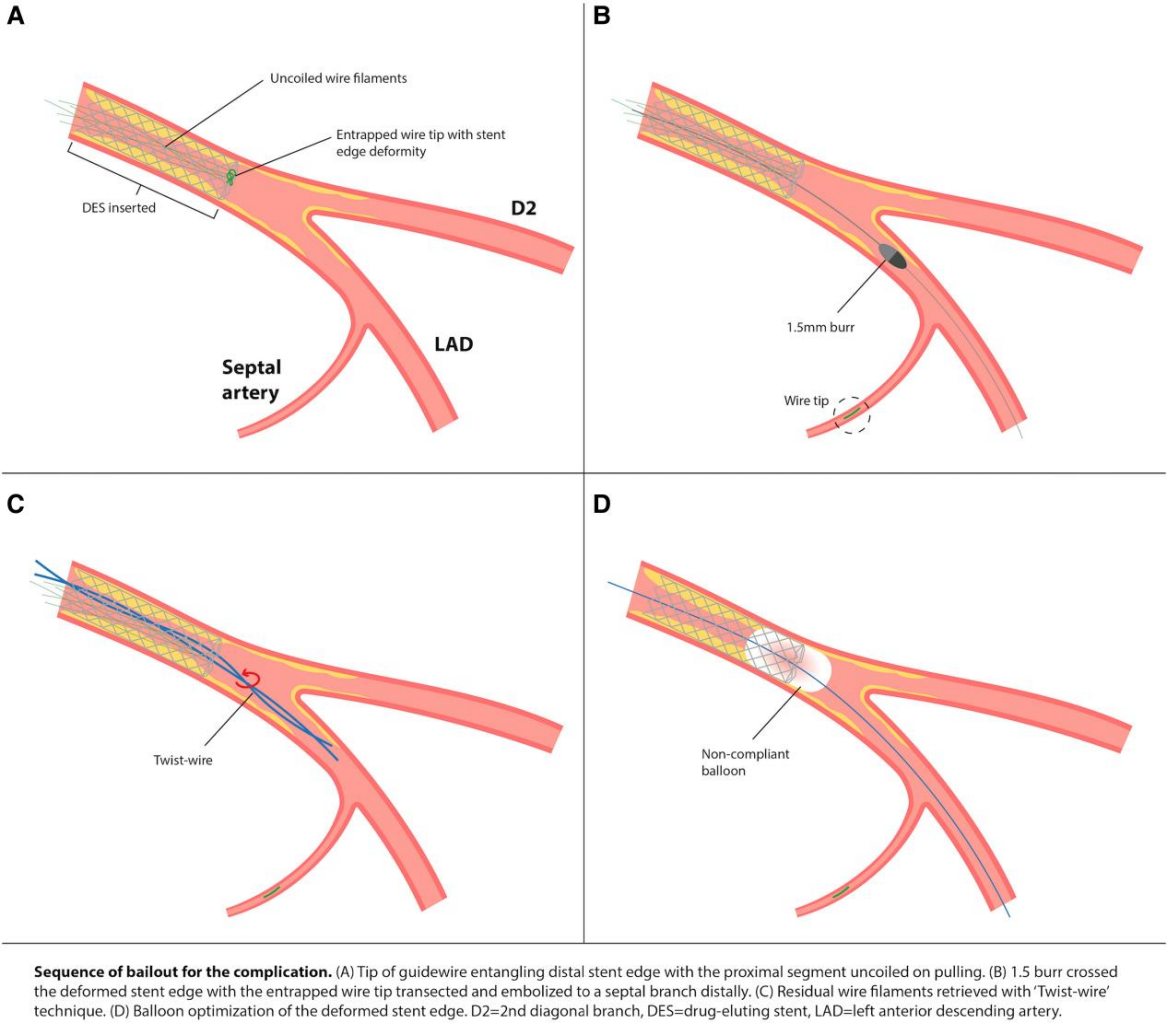


## Case presentation

**Figure 1:**
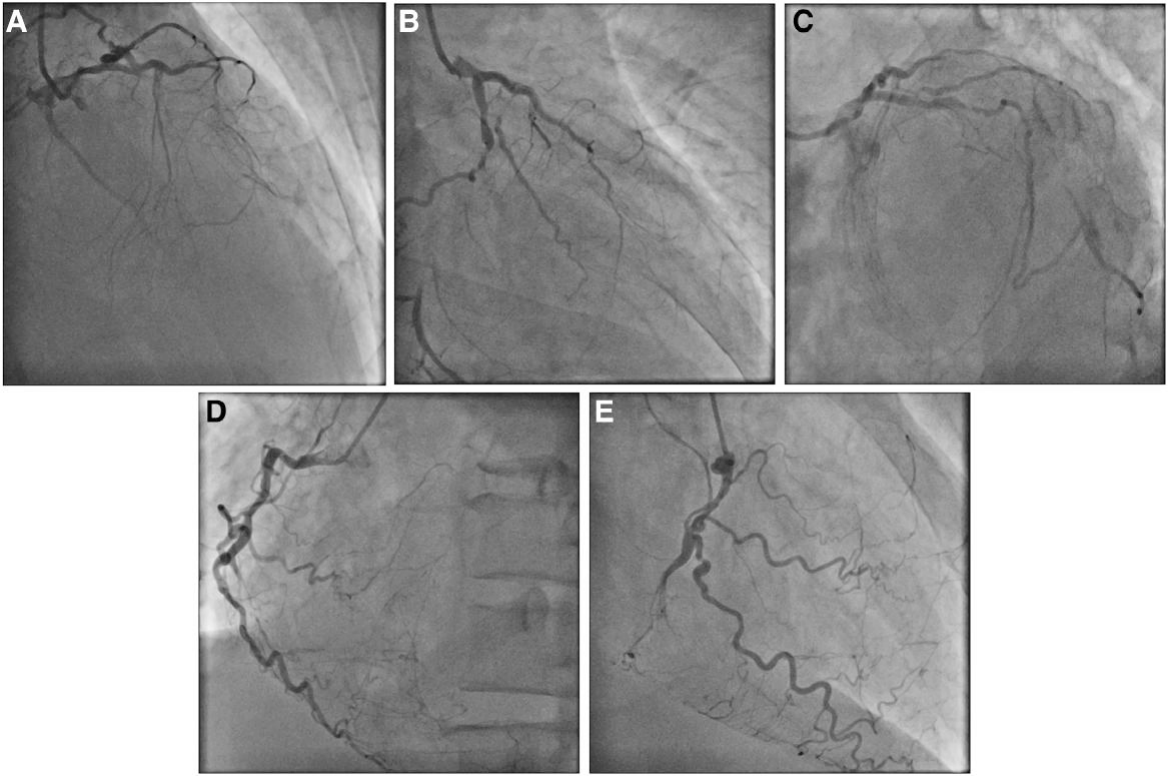
Diagnostic coronary angiography. (*A*) Right anterior oblique (RAO) cranial, (*B*) RAO caudal, and (*C*) left anterior oblique (LAO) caudal views of left coronary artery. (*D*) LAO and (*E*) RAO views of right coronary artery.

A 50-year-old man was a chronic smoker and suffered from a longstanding history of type 2 diabetes mellitus. He was admitted for decompensated heart failure as evidenced by pulmonary rales at bilateral lung bases, an elevated jugular venous pressure and bilateral lower limbs pitting oedema. Echocardiography showed severe left ventricular impairment with markedly reduced left ventricular ejection fraction of 20% and preserved myocardial thickness and contractility only in territory of left anterior descending artery (LAD). After initial stabilization of heart failure with intravenous diuretics, coronary angiography was performed showing severe left mainstem (LM) disease with collateralizing triple-vessel chronic total occlusion (CTO) (*Figure 1A–E*, Supplementary material online, *Videos S1* and *S2*). The calculated Society of Thoracic Surgeons (STS) operative risk score was 8.5% and coronary artery bypass graft was declined by cardiothoracic surgeons in view of high perioperative morbidity and mortality. Protected PCI under Impella CP (Abiomed, USA) support was arranged to revascularize the LAD first as it was the likely single viable territory from echocardiographic findings.

**Figure 2:**
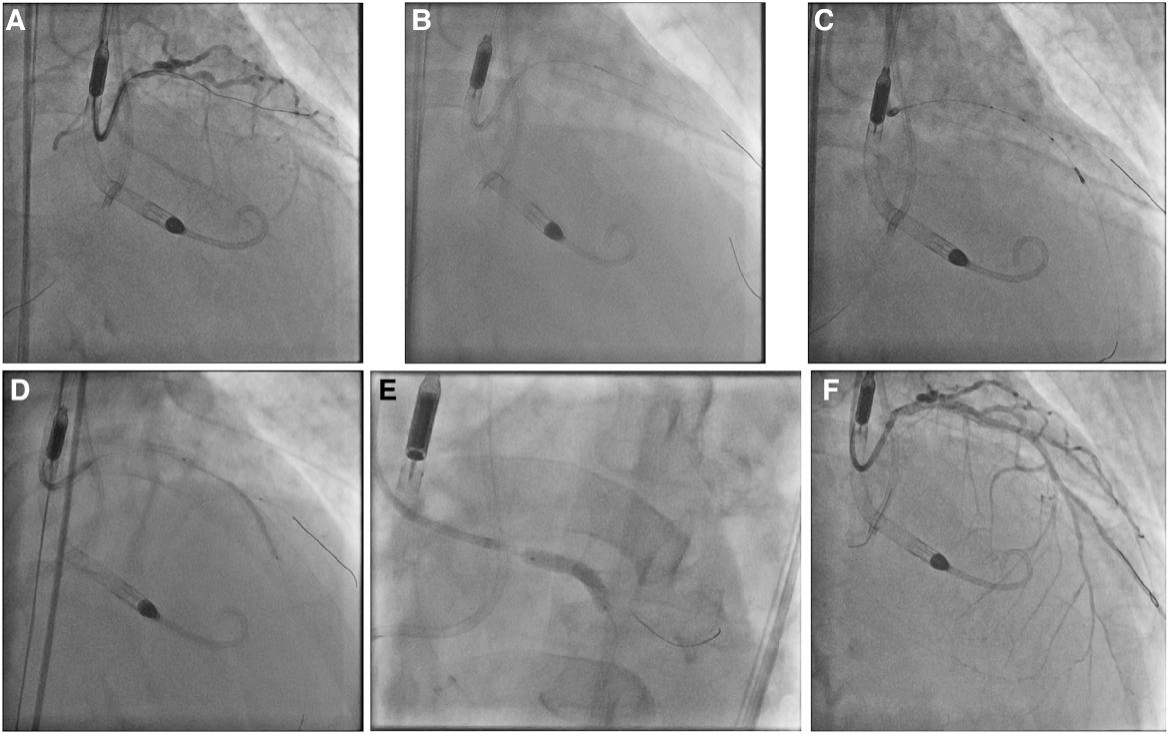
Percutaneous coronary intervention (PCI) of left anterior descending artery (LAD) chronic total occlusion (CTO). (*A*) Gaia Next 1 crossed LAD CTO and passed to 1st diagonal branch. (*B*) Sasuke assisted wiring of distal LAD with Runthrough Floppy wire. (*C*) Intravascular ultrasound (IVUS) interrogation to guide stent implantation. (*D*) 2.25 × 40 mm drug-eluting stent (DES) inserted to proximal and middle LAD. (*E*) 3.5 × 28 mm DES inserted to ostial left main (LM) and proximal LAD. (*F*) Good angiographic results after LM and LAD stenting and post-dilatation.

A 14 Fr Impella sheath was inserted to right femoral artery, through which an Impella CP was inserted for haemodynamic support. A 7 Fr Launcher (Medtronic, USA) EBU 3.5 guide catheter was inserted via right radial approach. Chronic total occlusion at proximal LAD was crossed with a Gaia Next 1 (Asahi Intecc, Japan) wire loaded on Turnpike microcatheter (Teleflex, USA) and passed to the first diagonal (D1) branch (*Figure 2A*), which was subsequently exchanged to a Runthrough Floppy (Terumo, Japan) wire. Distal part of the LAD was then wired with another Runthrough Floppy guidewire with the support of a Sasuke (Asahi Intecc, Japan) dual-lumen catheter (*Figure 2B*). Balloon angioplasty was performed followed by intravascular ultrasound (IVUS) assessment for lesion interrogation and determination of landing zones along the wire in distal LAD (*Figure 2C*). A 2.25 × 40 mm Orsiro Mission (Biotronik, Germany) drug-eluting stent (DES) was placed from proximal to middle part of LAD, just proximal to the take-off of second diagonal (D2) branch, while the D1 was protected by jailed balloon technique with a 2.0 mm semi-compliant balloon (*Figure 2D*). Another overlapping 3.5 × 28 mm Ultimaster Tansei (Terumo, Japan) DES was placed from ostial LM to proximal LAD segment, with wire protection of the left circumflex artery (LCX) (*Figure 2E*). Wire exchange was performed. The LCX was rewired with the original LAD wire and the jailed LCX wire was removed and advanced across the stented LM and LAD segments to the D2 branch, with a looped distal wire segment. This was followed by post-dilatation of stents with appropriately sized balloons. Subsequent IVUS demonstrated good stent expansion and apposition with satisfactory angiographic results (*Figure 2F*).

**Figure 3:**
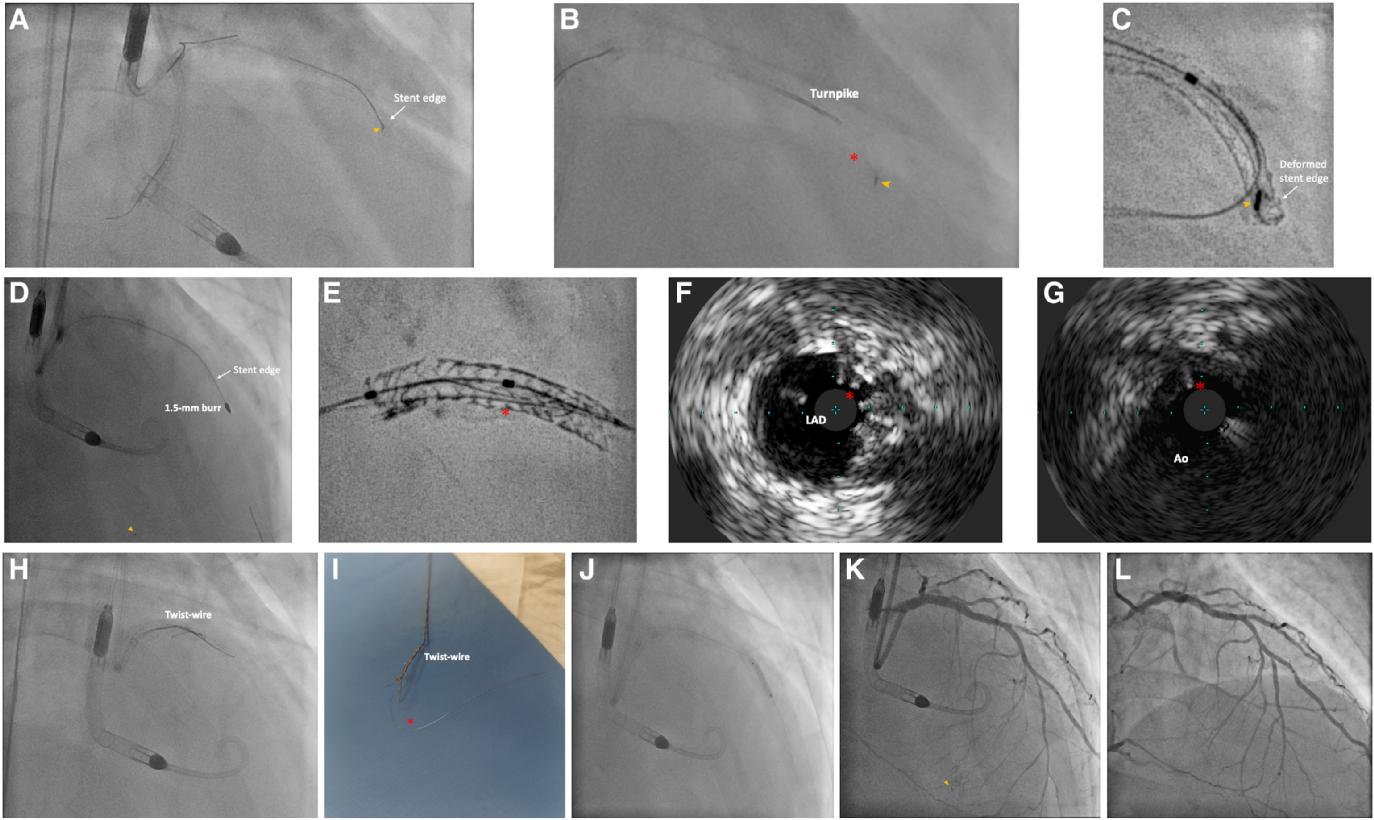
Bailout for entrapment and uncoiling of coronary guidewire. (*A*) Entrapment of coronary guidewire tip (arrowhead) during its removal. (*B*) Retrieval with Turnpike microcatheter (Teleflex, USA) led to unravelling (asterisk) of the distal segment of the guidewire from its tip (arrowhead). (*C*) ClearStent (Siemens Healthineers, Germany) showing the entrapped wire tip (arrowhead) with deformity of the distal stent edge. (*D*) Rotational atherectomy with 1.5 mm burr successfully crossed the deformed distal stent edge, with simultaneous transection and embolization of the radio-opaque wire tip (arrowhead) into a septal artery. (*E*) ClearStent showing residual wire filaments (asterisk) within the stent. (*F*) IVUS showing residual wire filaments (asterisk) in LAD stent. (*G*) Wire filaments (asterisk) extending to aorta (Ao). (*H*) Removal of wire filaments with ‘Twist-wire’ technique. (*I*) Successful retrieval of wire filaments (asterisk). (*J*) Balloon optimization of deformed distal stent edge. (*K*) Satisfactory final angiographic result, embolized wire tip confirmed to be in small septal artery (arrowhead). (*L*) Follow-up angiography at 4 months showing patent stents.

Upon removal of the Runthrough Floppy guidewire from D2, the wire tip got entangled with the distal stent edge (*Figure 3A*). Further retraction of the guidewire with a Turnpike microcatheter led to fracture of jailed guidewire and allowed only partial retrieval of its proximal part. The residual distal wire segment was left unravelled within the coronary bed and the struts at distal stent edge were also deformed precluding device passage even with a 1.0 × 5 mm semi-compliant balloon (*Figure 3B* and *C*; Supplementary material online, *Video S3*). A ‘Ping-pong’ guiding system was set up with another Launcher 7 Fr EBU 3.5 guide catheter inserted via the sheath of Impella CP. A Rotawire (Boston Scientific, USA) was delivered to distal LAD with difficulty. Rotational atherectomy (RA) with 1.5 mm burr was performed with an aim to clear the obstructing scaffolds. It successfully crossed the deformed distal stent edge, during which the uncoiled guidewire fragment was also transected with the radio-opaque distal tip embolized to a small septal artery (*Figure 3D*; Supplementary material online, *Videos S4* and *S5*).

Subsequent IVUS and ‘ClearStent’ (Siemens Healthineers, Germany) confirmed a residual portion of wire filaments extending from middle LAD to the aorta (*Figure 3E–G*; Supplementary material online, *Video S6*). They were then successfully removed with ‘Twist-wire’ technique with two Rinato Prowater (Asahi Intecc, Japan) guidewires (*Figure 3H* and *I*; Supplementary material online, *Video S7*). Further sequential post-dilatation of the deformed stent was done (*Figure 3J*). Final IVUS and angiography confirmed no significant residual stent deformity or wire filaments (*Figure 3K*; Supplementary material online, *Videos S8* and *S9*). Follow-up angiography at 4 months showed excellent results with widely patent stents (*Figure 3L*, Supplementary material online, *Video S10*).

## Discussion

Previous case report had demonstrated the effectiveness of RA in cutting the stretched spring wire after a core wire fracture.^5^ Rotational atherectomy in our case helped to (1) transect the uncoiled wire filaments for subsequent retrieval and (2) channel across the deformed and balloon uncrossable distal stent edge.

‘Twist-wire’ technique was widely reported for retrieval of fractured guidewire fragments.^6^ In our case, the retained uncoiled filaments were completely invisible on fluoroscopy after transection and embolization of the radio-opaque portion. Here we reported the first use of IVUS and stent enhancement system for localization of the wire filaments and guiding successful retrieval with ‘Twist-wire’ technique. This prevented surgical removal or additional stent placement for trapping the wire filaments, which carried substantial risk of future in-stent restenosis or thrombosis. The Volcano Eagle Eye Platinum IVUS (Philips, USA) was used, which was the only available IVUS catheter in our centre by the time of this case. A higher frequency rotational IVUS catheter, with a higher resolution, would allow better visualization of the uncoiled wire filaments. However, we do not recommend the use of optical coherence tomography (OCT) for imaging guidance in this case as wire filaments extending beyond the coronary ostium were unlikely to be visualized with OCT.

**Figure 4:**
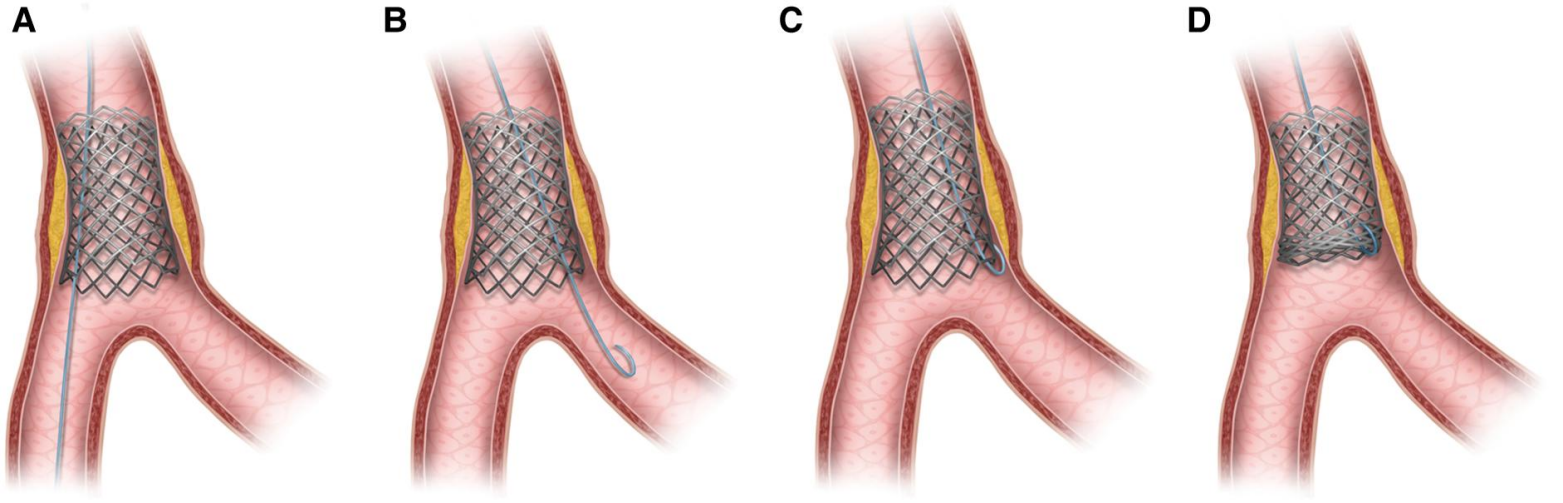
Schematic image depicting wire entrapment caused by ‘branch point malapposition’ (BPM). (*A*) Stent deployment just proximal to bifurcation along the wire in distal main vessel. (*B*) Wire switched to side branch with a loop tip. (*C*) Wire tip caught by distal stent edge during retrieval due to BPM. (*D*) Stent deformity caused by pulling of entrapped wire.

**Figure 5:**
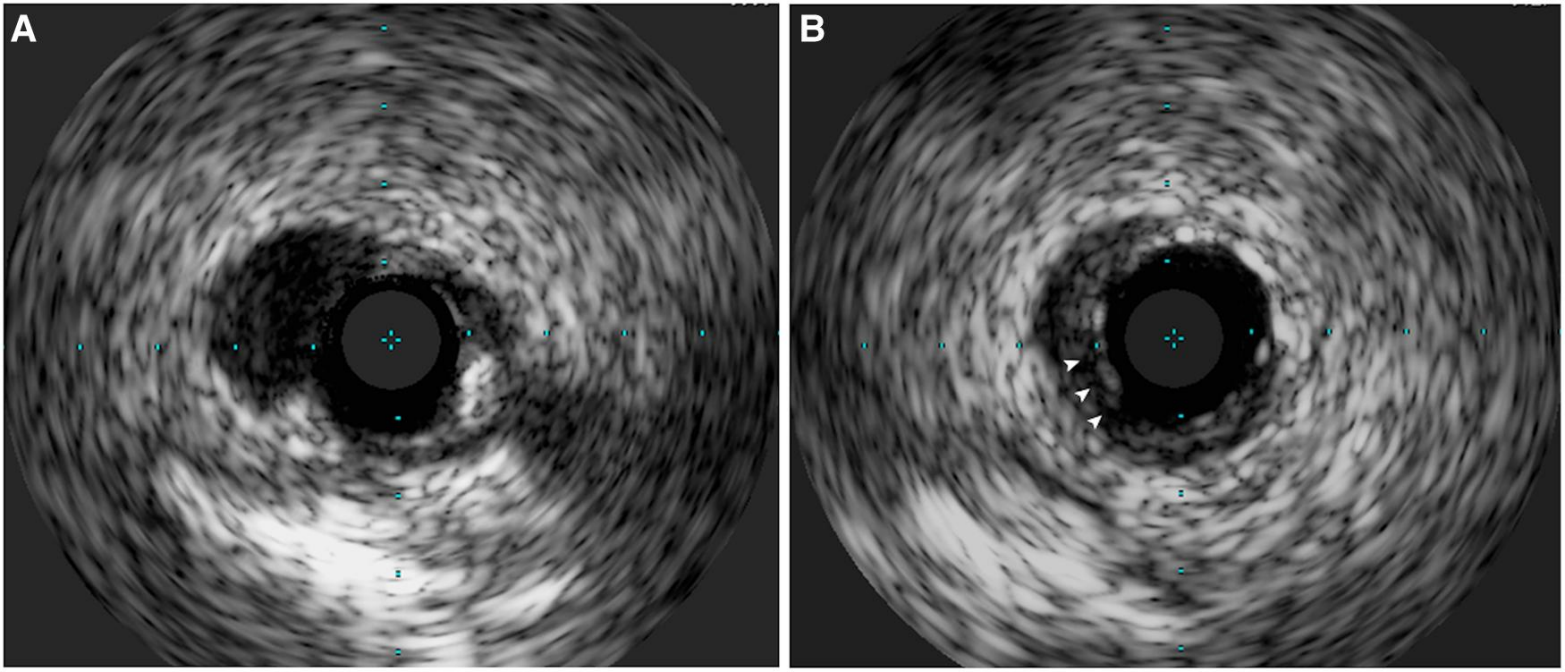
IVUS image demonstrating BPM. (*A*) Absence of stent coverage at carina. (*B*) Stent coverage at branch point with potentially malapposed struts (arrowheads).

This case also reported an unusual circumstance of wire entrapment, without any wire jailing behind stent struts as confirmed by IVUS assessment post-stenting. Here we proposed the phenomenon of ‘branch point protrusion’ (BPP) being the likely mechanism causing wire entrapment. During stent implantation, the middle LAD stent was initially inserted along the wire placed in distal main vessel (MV) (*Figure 4A*). Yet after subsequent stent insertion, wire in distal MV was switched to a side branch (SB) with a looped tip (*Figure 4B*). During wire withdrawal, the distal wire loop may inadvertently get caught by the distal stent edge at the branch point if the stent is slightly protruded into the area of confluence in the bifurcation and not well apposed in this region (*Figure 4C*). Further forceful pulling of the entrapped wire then resulted in deformity of the stent edge (*Figure 4D*). In our case, BPP was so trivial that its detection can be difficult even with the use of IVUS (*Figure 5A* and *B*). Furthermore, wire tip fatigue and microdamage, either due to prolonged procedure or prior jailing for side branch protection, predisposed to trapping and entanglement at stent edge alongside BPP.

## Conclusion

Our case was the first to highlight the combined use of IVUS and stent enhancement system to localize fluoroscopically invisible uncoiled wire filaments, guiding complete retrieval with ‘Twist-wire’ technique. In addition, interventional cardiologists should be aware of BPP of stents that, although subtle on intracoronary imaging, may result in device entrapment and stent edge compromise.

## Supplementary Material

ytae258_Supplementary_Data

## Data Availability

Anonymized clinical data will be available from the corresponding author for reasonable requests after seeking institutional approval for 3 years from date of publication.

## Lead author biography

Dr Siu-Fung Wong was born in Hong Kong in 1992 and graduated from the Chinese University of Hong Kong in 2015. He was trained in cardiology at Prince of Wales Hospital and United Christian Hospital. He is currently working at United Christian Hospital as interventional cardiologist. He specializes in complex cardiovascular interventions.

## Supplementary material

**Consent:** The authors confirm that written consent for the submission and publication of this case report, including images and associated text, has been obtained from the patient, following COPE guidance.

**Funding:** None.

**Ethics approval:** Ethical approval from institution was not required as the submitted work is case report.
